# Investigating young-adult social outcomes of attention deficit hyperactivity disorder

**DOI:** 10.4088/jcp.22m14379

**Published:** 2023-01-25

**Authors:** Lucy Riglin, Andrew Todd, Rachel Blakey, Amy Shakeshaft, Evie Stergiakouli, George Davey Smith, Kate Tilling, Anita Thapar

**Affiliations:** 1 Division of Psychological Medicine and Clinical Neurosciences, MRC Centre for Neuropsychiatric Genetics and Genomics, Cardiff University, UK; 2 Population Health Sciences and MRC Integrative Epidemiology Unit, University of Bristol, Bristol, UK

**Keywords:** ADHD, young-adult, persistence, social outcomes, ALSPAC

## Abstract

**Objective:**

Attention Deficit Hyperactivity Disorder (ADHD) is associated with a range of adverse outcomes in adult life. However it is unclear whether the risk pathways to adverse adult outcomes are established during childhood or whether associations are driven by concurrent ADHD symptoms that have persisted to adulthood.

**Methods:**

We examined associations between broadly defined child-limited (remitted) and persistent ADHD (assessed using the ADHD subscale of the Strengths and Difficulties Questionnaire) with social outcomes (low emotional and instrumental support, antisocial behaviour, employment, receipt of state benefits as an indicator of socio-economic disadvantage, homelessness) at age 25 years in a UK longitudinal population sample ALPSAC (the Avon Longitudinal Study of Parents and Children, age 25 data collected between years 2017 and 2018): total N=6439.

**Results:**

Up to 20% of young-people with less favourable social outcomes at age 25 had persistent ADHD. Persistent ADHD was associated with an increased likelihood of being not in education, employment or training (NEET: OR=3.71, 95% CI=2.06 to 6.67, p=1x10^-05^) and receiving state benefits (OR=2.72, 95% CI=1.62 to 4.57, p=2x10^-04^) at age 25 years compared to those without ADHD. We did not find strong evidence of associations between child-limited ADHD and social outcomes (NEET OR=1.20, 95% CI=0.54 to 2.69, p=0.65; state benefits OR=1.38, 95% CI=0.76 to 2.51, p=0.29). Persistent ADHD associations with negative social outcomes were observed across family-of-origin income groups, sex and were not explained by comorbidity.

**Conclusion:**

Our findings highlight the importance of continued monitoring and management of ADHD symptoms and related social as well as clinical outcomes across development into adulthood. Future research is needed to identify what factors promote positive social outcomes, including effective treatment of adult ADHD symptoms.

## Introduction

Attention Deficit Hyperactivity Disorder (ADHD) is a highly heritable neurodevelopmental disorder with typical onset in childhood. Although most affected individuals continue to display some ADHD symptoms and impairment after childhood, others remit before adult life.^1, 2^ ADHD, whether defined categorically as a clinical disorder or as continuously distributed total symptom scores, is associated with multiple adverse adult clinical outcomes such as depression, anxiety, substance misuse and self-harm as well as worse social, occupational and physical health outcomes than those who are unaffected.^3–6^ Many of these adversities carry great personal, familial and societal cost.

The association between ADHD and higher levels of antisocial behaviour is well established^7^. One meta-analysis estimated fivefold higher prevalence of people with ADHD detained in youth prison populations (30.1%) and tenfold in adult prison populations (26.2%) across a number of countries, compared to general population prevalence estimates at equivalent ages.^8^ ADHD is also associated with an increased likelihood of not completing secondary school or attending tertiary education,^9^ employment difficulties, homelessness and financial dependence on parents or government/public assistance in adulthood.^10, 11^ Finally, studies also suggest an association between ADHD and poorer quality social relationships and support in adulthood.^12, 13^

A variety of different research designs have been used to infer causal relationships between ADHD and several different clinical and physical health outcomes; these include time-series investigations of prescription data and Mendelian randomisation approaches. These studies suggest that the relationships between ADHD and some adverse outcomes, including depression,^14, 15^ substance misuse, cigarette smoking, BMI and coronary artery disease^16^ may be causal, arising as a direct result of ADHD. If that is the case, then vigorous and effective support/treatment of ADHD would be crucial for preventing adverse adult outcomes.

For many individuals however, the transition from child to adult mental health services disrupts engagement with clinicians and treatment continuity. That may not be a problem if ADHD has remitted and adverse adult outcomes primarily are influenced by concurrent ADHD symptoms. However, if ADHD symptoms in childhood lead to adversities or disadvantages (e.g. victimization, maltreatment and educational failure) across childhood and adolescence, then the risk mechanisms leading to poor adult outcomes have already been set in place. That would support the argument for continued monitoring of those with a history of childhood ADHD even if ADHD symptoms have declined or remitted. One previous study supports the latter hypothesis. In the UK prospective twin cohort E-risk, those with remitted ADHD showed worse outcomes at age 18 years than those who have never had ADHD but not to the same extent as those with persistent ADHD.^17^ The question of what happens to those with ADHD that remits is clinically important: if ADHD appears to have remitted by late adolescence, should these young people be discharged from services or continue to be monitored by adult mental health or primary care services even if this follow-up is provided less frequently than for those with persistent ADHD?

In this study we utilise a UK longitudinal birth cohort, followed up to age 25 years to address this question. We set out to examine the adult social outcomes at age 25 years associated with broadly defined ADHD, differentiating those that were child-limited (remitted) and persistent. We hypothesised that those with ADHD who had remitted by age 25 years, as well as those with persistent ADHD, would show adverse social outcomes by age 25 years.

## Materials and methods

### Sample

We analysed data from the Avon Longitudinal Study of Parents and Children (ALSPAC), a well-established prospective, longitudinal UK birth cohort study.^18–20^ Pregnant women resident in Avon, UK with expected dates of delivery 1st April 1991 to 31st December 1992 were invited to take part in the study. The initial number of pregnancies enrolled is 14,541 (for these at least one questionnaire has been returned or a “Children in Focus” clinic had been attended by 19/07/99). Of these initial pregnancies, there was a total of 14,676 foetuses, resulting in 14,062 live births and 13,988 children who were alive at 1 year of age. When the oldest children were approximately 7 years of age, an attempt was made to bolster the initial sample with eligible cases who had failed to join the study originally. As a result, the total sample size for data collected after the age of seven is therefore 15,454 pregnancies, resulting in 15,589 foetuses. Of these 14,901 were alive at 1 year of age. Part of this data was collected and managed using REDCap electronic data capture tools hosted at the University of Bristol.^21^ REDCap (Research Electronic Data Capture) is a secure, web-based software platform designed to support data capture for research studies. Ethical approval for the study was obtained from the ALSPAC Law and Ethics Committee and Local Research Ethics Committees. Informed consent for the use of data collected via questionnaires and clinics was obtained from participants following the recommendations of the ALSPAC Ethics and Law Committee at the time. Consent for biological samples has been collected in accordance with the Human Tissue Act (2004). Please note that the study website contains details of all the data that is available through a fully searchable data dictionary and variable search tool: http://www.bristol.ac.uk/alspac/researchers/our-data/. Further details of the study, measures and sample can be found elsewhere.^18–20^ Where families included multiple births, we included the oldest sibling.

### ADHD

**Figure 1:**
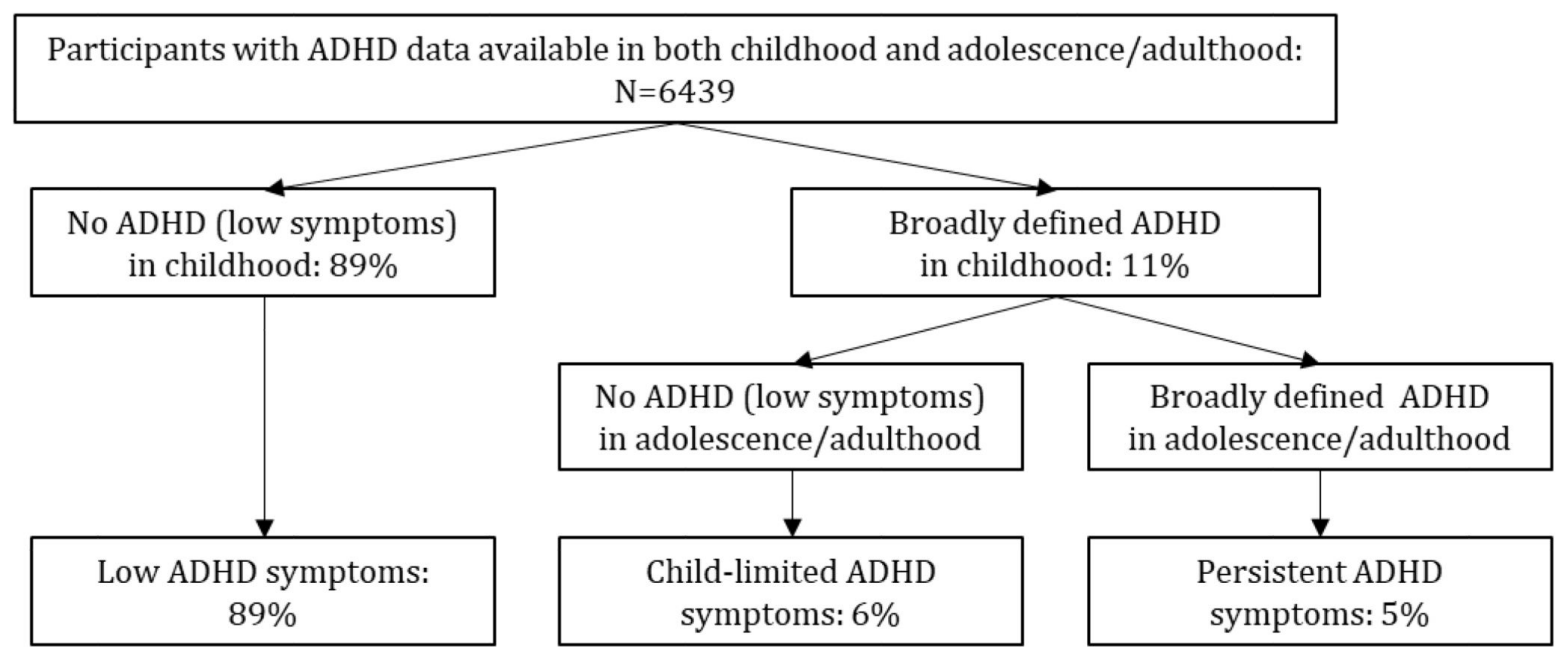
ADHD groups for primary analyses

ADHD symptoms were assessed using the 5-item ADHD subscale of the Strengths and Difficulties Questionnaire (SDQ: possible range 0-10),^22^ a well-established screening measure completed by parents about their children at approximately ages 7, 8, 9, 12 and 17, and by self-report at age 25 years. The SDQ ADHD subscale has high validity in identifying DSM-5 ADHD caseness. The category of broadly defined ADHD was applied to participants who scored above the recommended SDQ cut-point for identifying ADHD diagnosis. These cut-points are ≥8 in childhood and adolescence^21^ and ≥5 for self-reports at age 25 years.^23^ In-line with previous work^24^ childhood ADHD was defined as the presence of broadly-defined ADHD at ages 7, 8, 9 or 12 and late adolescent/young-adult ADHD where participants scored above the SDQ-ADHD subscale cut-point at ages 17 or 25. Based on these categories, individuals were categorised as having low ADHD levels (i.e. below the SDQ cut-point in both childhood and late adolescence/young-adulthood), child-limited ADHD (i.e. above the SDQ cut-point in childhood but not in late adolescence/young-adulthood) or persistent ADHD (i.e. above the SDQ cut-point in both childhood and late adolescence/young-adulthood). These groups are shown in Figure 1. Sensitivity analyses were conducted investigating “late-onset” ADHD (ADHD symptoms first manifest after the age of 12 years) and are available on request from the author.

### Social outcomes

Social outcomes were assessed based on self-reports at age 25 years. *Emotional* (e.g. having someone to talk to) and *instrumental support* (e.g. having someone who can provide practical support) were measured by the National Institute of Health (NIH) Toolbox Adult Social Relationship Scales^25^ (possible ranges 0-32). Low support was defined as the bottom 10% for descriptive purposes. *Aggressive and non-aggressive antisocial behaviour* were measured using the Edinburgh Study of Youth Transitions and Crime^26^ which assessed engagement in 12 antisocial activities in the past year (possible range 0-12). Antisocial behaviour was defined as engagement in any of the relevant anti-social activities: the aggressive (4 items) and non-aggressive (8 items) antisocial behaviours (details available on request from the author). *Not in Education, Employment or Training (NEET) status* was derived in-line with the UK Office for National Statistics definition^27^ (details available on request from the author). *State benefit recipient* was defined as receiving unemployment-related benefits, income support, sickness or disability benefits, housing benefits (including council tax benefit, rent or rate rebate) or tax credits. We included receiving state benefits as an outcome as a proxy measure of socio-economic disadvantage. *Homelessness* was assessed using one item from a 27-event checklist that asked about events experienced in the previous 12 months.

### Variables for sensitivity analyses

Sensitivity analyses were conducted stratifying by sex and family of origin (“family”) income. *Family income* was measured by mother-report at approximately child age 11 years as the average household income including social benefits each week on a 10-point scale from <£120 to ≥£800. Four income groups were generated, with lower/higher income defined based on falling below/above the median and the lowest and highest subsequently identified as bottom/top decile.

We also examined whether associations between ADHD and age 25 social outcomes were still present in the absence of child comorbidities. *Low IQ* was defined as IQ<80, measured using the Wechsler Intelligence Scale for Children.^28^ *Autism symptoms* were measured using the parentrated 12-item Social Communication Disorders Checklist (SCDC)^29^ at ages 7 years (cut-point ≥9). *Conduct problems* were assessed using the parent-rated 5-item SDQ^22^ subscale at age 7 years (cut-point ≥4). *Emotional problems* were also assessed using the parent-rated 5-item SDQ^22^ subscale at age 7 years (cut-point ≥5).

Finally, we examined whether associations between ADHD and age 25 social outcomes were still present in the absence of young-adult emotional problems and when excluding those with ADHD medication use. *Young-adult emotional problems* were also assessed using the self-rated 5-item SDQ^22^ subscale at age 25 years (cut-point ≥6). *Lifetime ADHD medication* use (methylphenidate, dexamfetamine or atomoxetine) was assessed by self-report at age 25 years.

### Analyses

Analyses were conducted using multiple imputation with inverse probability weighting (IPW/MI),^30^ including individuals with ADHD data available in both childhood and adolescence/adulthood (N=6439): social outcome data were available for N=3591-3654 depending on the variable (details available on request from the author). We used logistic and linear regression to examine associations with social outcomes, with low ADHD symptoms as the reference group to estimate associations for child-limited and persistent ADHD. Sensitivity analyses examined associations stratified by sex, family income, childhood comorbidities (low IQ, autism symptoms, conduct problems and emotional problems), young-adult emotional problems and excluding those with ADHD medication use. Sensitivity analyses based on MI (without IPW), IPW (without MI) and complete-case analyses are available on request from the author. Finally, sensitivity analyses using different definitions of ADHD were conducted (a) defining childhood ADHD as having high ADHD symptoms at multiple times across 7, 8, 9 or 12 (i.e. at least twice, rather than at least once), (b) defining high ADHD symptoms at age 25 years using the more stringent cut-point of ≥6 ^23^ and (c) defining persistent ADHD based on the presence of high symptoms at age 25 years only (i.e. rather than age 17 or 25 years). Sensitivity analyses using different definitions of ADHD are also available on request from the author.

## Results

Approximately 89% of the sample were defined as having low ADHD symptom levels, 6% as having child-limited ADHD symptoms and 5% as having persistent symptoms.

Estimated social outcome means/proportions by ADHD group are shown in Table 1. The proportions of individuals with ADHD by outcome (e.g. of those with low emotional support, the proportion with child-limited and persistent ADHD) are shown in Table 2: up to 20% of those showing less favourable adult outcomes such as being NEET, receiving state benefits and being homeless had ADHD.

As shown in Table 3, persistent ADHD was associated with lower adult emotional support and an increased likelihood of being NEET and of receiving state benefits compared to those with low ADHD symptoms. There was a trend for persistent ADHD to be associated with antisocial behaviour (aggressive and non-aggressive) and homelessness; we did not find strong evidence of association with lower instrumental support for persistent ADHD. There was also not strong evidence of association for child-limited ADHD with any of the social outcomes at age 25 years.

**Figure 2:**
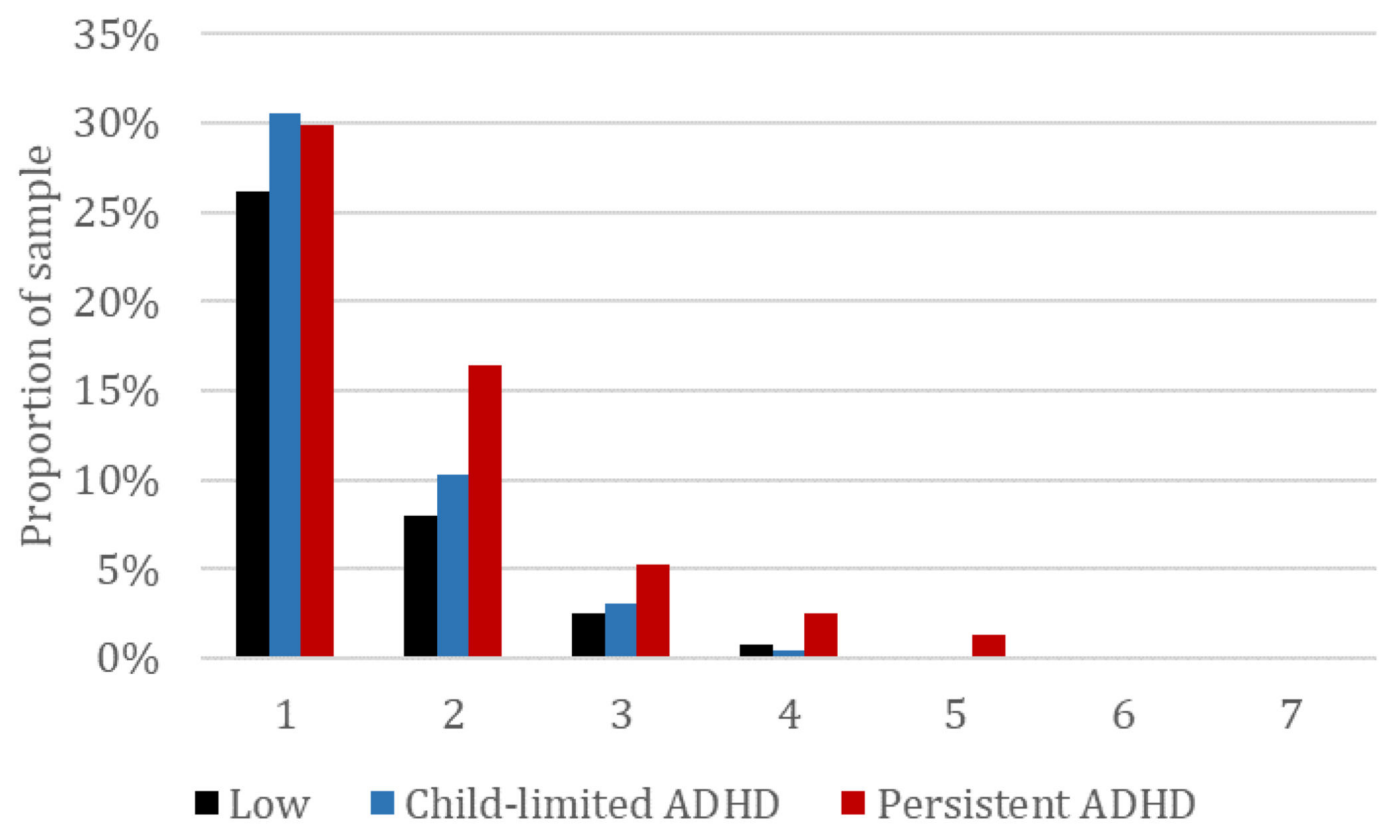
Total number of social outcomes by ADHD group using IPW/MI to account for missing data Abbreviations: IPW/MI = multiple imputation with inverse probability weighting

Persistent ADHD was also associated with a higher total number of these adverse social outcomes (defined categorically) compared to those with low ADHD symptoms (OR=2.07, 95% CI=1.47 to 2.93, p=4x10-^05^), whereas evidence of association for child-limited ADHD was weaker (OR=1.33, 95% CI=0.93 to 1.89, p=0.11). The estimated total number of these outcomes by ADHD group are shown in Figure 2: for those with persistent ADHD, 45% were estimated to have none of the adverse social outcomes assessed at age 25, 30% to have one and 25% to have multiple (this was 56%, 31% and 14% respectively for those with child-limited ADHD and 63%, 26% and 11% for those with low ADHD symptoms).

### Sensitivity analyses

Sensitivity analyses results are available on request from the author.

Analyses stratified by sex found associations to be generally consistent across males and females, with some evidence that associations for persistent ADHD with lower emotional support may be driven by females and association with NEET status driven by males (although confidence intervals overlapped). Associations stratified by family of origin income also found associations to be generally consistent across income levels, with overlapping confidence intervals across groups.

Sensitivity analyses also examined whether associations between ADHD and adult social outcomes were still present in the absence of child comorbidities (low IQ, autism symptoms, conduct problems and emotional problems), young-adult emotional problems and ADHD medication use. Evidence of association between persistent ADHD and lower emotional support, being NEET and receiving state benefits remained when excluding these child comorbidities, young-adult emotional problems and taking ADHD medication although the association with state benefits was somewhat attenuated when excluding those with childhood ASD symptoms and association with lower emotional support was somewhat attenuated when excluding those taking ADHD medication.

Missing data sensitivity analyses showed a similar pattern of results across different approaches.

Finally, sensitivity analyses using different definitions of ADHD also showed a similar pattern of results, as did those investigating “late-onset” ADHD.

## Discussion

This study set out to examine the adult social outcomes at age 25 of ADHD symptoms across childhood and young-adulthood in a longitudinal birth cohort, differentiating those that were child-limited (remitted) and persistent. The findings observed did not support our hypothesis that adults with both remitted and persistent ADHD would show adverse social outcomes by age 25 years. While we found associations with persistent ADHD, we did not find such strong evidence of association with child-limited ADHD.

We found ADHD that persisted into young-adulthood was associated with multiple adverse social outcomes including lower levels of emotional support and an increased likelihood of being NEET (Not in Education, Employment or Training) and of receiving state benefits (a proxy measure of socio-economic disadvantage). Approximately 20% of young people with NEET status, receiving state benefits and who were homeless had ADHD. This pattern of associations was present across sex, family of origin income level, and when excluding childhood comorbidities, young-adult emotional problems and ADHD medication use. While it is beyond the scope of this paper to investigate the mechanisms linking (persistent) ADHD to these social outcomes, possible explanations include ADHD-related core symptoms and emotional regulation difficulties impacting on the initiation and/or maintenance of supportive relationships and upon educational attainment. Interestingly we did not find strong evidence of association between persistent ADHD and lower instrumental support, which may result from a reliance on (and therefore increase use of) others for practical support. Difficulties in ADHD-service engagement (including during the transition from child to adult services) at a time of formal examinations, transitions out of school and future planning may also contribute to poor social outcomes. We also found persistent ADHD to be associated with an increased number of these negative social outcomes even though the sample is a population-based cohort and we defined ADHD broadly. Around 1 in 4 of this group had more than one of these negative social outcomes. Future research needs to identify what modifiable social factors promote better social outcomes. There also was some evidence that women with ADHD may be more likely to have impairments in social relationships (low emotional support) and men with ADHD to have impairments in employment/training (NEET). Further work would be needed to investigate potentially different pathways for men and women between ADHD and social outcomes.

Our findings suggest that directing resources to supporting those with ADHD and monitoring symptoms across adolescence and young-adulthood may be a beneficial area of focus. This age captures a period of increasing socio-occupational, personal and financial demands, when young people typically graduate from education into the world of further training and/or employment. The absence of strong evidence of association between child-limited ADHD suggests that the risk-mechanisms linking ADHD and social outcomes in young-adulthood are not exclusively set in place in childhood – that effective ADHD treatment and other interventions that reduce symptoms may help prevent adverse adult outcomes. Indeed, previous work has found for example that stimulant medication in young people with ADHD is associated with a decreased risk of subsequent smoking and substance use disorders in adolescence (at 5-year follow-up)^31^: but that this effect may not persist into young-adulthood (10-yearfollow-up).^32^ However our study design cannot differentiate whether associations with outcomes are a causal consequence of ADHD symptoms. For example, evidence suggests that ADHD persistence is associated with ADHD severity and a higher genetic loading than remitted ADHD.^33^ Also ADHD symptom persistence into young-adulthood does not exclude the possibility that risk mechanisms for social outcomes were set in place earlier in development for this group. Regardless, our findings highlight the importance of ADHD monitoring and management of ADHD symptoms across development, the transition from child to adult services and the variability in adult social outcomes.

Our findings somewhat differ from a previous UK cohort study which showed association between remitted ADHD and life satisfaction, NEET status and criminal convictions (although not social isolation) at the age of 18 years.^17^ One explanation is that our study focussed on broadly defined ADHD whereas the previous work^17^ examined ADHD diagnosis, which means that individuals who were categorised as having remitted ADHD (i.e. did not meet diagnostic threshold at 18 years) may still have had (subthreshold) ADHD symptoms at 18.^17^ Such individuals would have been categorised as having persistent ADHD symptoms in our study. It could be that our (less common) outcomes were defined more stringently and were more severe. Alternatively, differences could be due to follow-up period: it may be that the familial, social, occupational and personal life events and changes that occur between ages 18 and 25 explain the apparent contrasting findings.

Our study findings should be considered in light of limitations. As with many longitudinal population-based samples, ALSPAC also suffers from non-random attrition, with those who drop out more likely to be at elevated risk of psychopathology.^34, 35^ We used multiple imputation with inverse probability weighting to try to minimise the effect of missingness and findings were consistent across different approaches. We focused on ADHD symptoms in a population cohort and although evidence suggests that ADHD behaves as a continuously distributed trait without clear-cut thresholds in terms of associations with adverse outcomes,^36^ our findings may not be able to be generalised to clinical diagnosis or those in clinical services. However our prevalence rates of ADHD are not much higher than the rate of ADHD diagnosis in many population surveys. The use of a population sample also limited our power for detecting association with rarer outcomes such as homelessness. Finally, beyond examining young-adult emotional problems we did not examine the potential impact of presence and treatment of additional comorbid psychiatric disorders in adulthood.

Further research using alternative designs such as those that assess causal inference is needed to test whether the associations we found are causal and work is needed to identify potentially modifiable risk mechanisms, groups that are at highest risk and factors that promote positive adult social outcomes. Identification of such moderating and mediating factors as well as those relating to service disengagement is needed to be able to better address the emotional wellbeing and social marginalisation of at-risk young people with ADHD. The effect of the COVID-19 global pandemic on the social outcomes of young people with ADHD is also as yet unknown: the associated additional challenges that this has posed on young people and understanding how they can be best supported as they progress into wider society remains vital.

The funding and expansion of support for young-adults with ADHD in educational/occupational establishments and benefit systems may help reduce negative outcomes. Holistic age-specific ADHD resources, interventions, support and services that span the lifespan may be beneficial in addressing the range of adverse outcomes associated with ADHD, as well as core features. Mental health awareness initiatives are increasingly taking place in educational establishments, however specific ADHD-informed interventions are indicated to anticipate and support young people in making choices around goals for further training and/or occupational activity. Understanding individuals with ADHD and matching strengths and interests (and acknowledging areas of challenge) with appropriate lines of work may serve to increase the likelihood of increased enjoyment, satisfaction and longer-term engagement in meaningful socio-occupational activity. This may in turn have positive effects on navigating supportive relationships, emotional wellbeing and use of maladaptive coping strategies. Early identification and specific careers guidance by resourced practitioners with increased awareness of ADHD would support and highlight to young people with persistent symptoms the wide range of complimentary, realistic and exciting future opportunities for their diverse skill sets.

In conclusion, our study found ADHD that persists to young-adulthood is associated with a range of less favourable social outcomes, including low emotional support, NEET status and receiving state benefits (as an indicator of socio-economic disadvantage) although these adverse outcomes were not inevitable. Strong evidence of association was not found for child-limited ADHD. These findings support the continued monitoring and management of ADHD across development and the transition from child to adult services, including in areas of functional impairment beyond core ADHD features.

## Figures and Tables

**Table 1:** Social outcomes means/percentages by ADHD group using IPW/MI to account for missing data

	Low	Child-limited ADHD	Persistent ADHD
Emotional support	43.04 (42.58 to 43.49)	42.44 (40.47 to 44.41)	39.07 (37.09 to 41.05)
Instrumental support	39.41 (38.80 to 40.02)	40.63 (38.31 to 42.95)	38.05 (35.54 to 40.56)
Aggressive ASB	5.11% (3.87 to 6.34)	7.05% (2.17 to 11.93)	10.04% (3.28 to 16.81)
Non-aggressive ASB	13.04% (11.64 to 14.44)	12.26% (6.42 to 17.68)	19.87% (12.24 to 27.49)
NEET	5.54% (4.44 to 6.64)	6.77% (2.00 to 11.53)	18.02% (9.95 to 26.08)
State benefit recipient	7.67% (6.52 to 8.83)	10.43% (5.04 to 15.83)	18.56% (10.86 to 26.27)
Homelessness	1.63% (0.82 to 2.45)	1.57% (-1.48 to 4.62)	4.65% (-0.04 to 9.71)

N=6439. 95% confidence intervals in parentheses.

Abbreviations: ASB = antisocial behaviour, IPW/MI = multiple imputation with inverse probability weighting, NEET = Not in Education, Employment or Training.

**Table 2:** Percentage of individuals with ADHD, by social outcome using IPW/MI to account for missing data

	Persistent ADHD	Child-limited ADHD
Low emotional support (10%)^a^	8.87% (5.54 to 12.20)	6.54% (3.22 to 9.85)
Low instrumental support (10%)^a^	5.40% (2.23 to 8.56)	5.47% (2.38 to 8.55)
Aggressive ASB (5%)	9.35% (3.27 to 15.44)	7.85% (2.57 to 13.14)
Non-aggressive ASB (13%)	7.62% (4.68 to 10.56)	5.63% (3.14 to 8.11)
NEET (6%)	14.78% (8.24 to 21.32)	6.58% (1.98 to 11.18)
State benefit recipient (8%)	13.34% (-0.66 to 27.33)	5.34% (-5.17 to 15.84)
Homelessness (2%)	12.29% (6.72 to 15.86)	7.61% (3.75 to 11.47)

N=6439.

a For continuous outcomes, low support defined as the bottom 10%. 95% confidence intervals in parentheses.

Abbreviations: ASB = antisocial behaviour, IPW/MI = multiple imputation with inverseprobability weighting, NEET = Not in Education, Employment or Training.

**Table 3:** Associations between ADHD group and social outcomes using IPW/MI to account for missing data

	Child-limited ADHD	Persistent ADHD
Emotional support	RC=-0.60 (-2.59 to 1.39), p=0.56	RC=-3.97 (-5.94 to -1.99), p=9x10^-05^
Instrumental support	RC=1.22 (-1.13 to 3.57), p=0.31	RC=-1.36 (-3.87 to 1.15), p=0.29
Aggressive ASB	OR=1.38 (0.65 to 2.93), p=0.40	OR=2.02 (0.95 to 4.30), p=0.07
Non-aggressive ASB	OR=0.92 (0.56 to 1.53), p=0.76	OR=1.64 (1.01 to 2.67), p=0.05
NEET	OR=1.20 (0.54 to 2.69), p=0.65	OR=3.71 (2.06 to 6.67), p=1x10^-05^
State benefit recipient	OR=1.38 (0.76 to 2.51), p=0.29	OR=2.72 (1.62 to 4.57), p=2x10^-04^
Homelessness	OR=0.74 (0.08 to 6.50), p=0.79	OR=2.81 (0.86 to 9.12), p=0.09

N=6439. Reference group = low ADHD group. 95% confidence intervals in parentheses.

Abbreviations: ASB = antisocial behaviour, IPW/MI = multiple imputation with inverse probability weighting, NEET = Not in Education, Employment or Training, OR = odds ratio, RC = regression coefficient.

## Clinical Points

- ADHD is associated with a range of adverse outcomes in adult life, but it is unclear whether this is driven by concurrent ADHD symptoms that have persisted to adulthood.
- Continued monitoring and management of ADHD into adulthood is important, including in areas of functional impairment beyond core ADHD features.
